# Diagnostic reference levels in interventional neuroradiology: a scoping review

**DOI:** 10.1007/s00330-026-12472-0

**Published:** 2026-03-25

**Authors:** Marvin Grech, Francis Zarb, Reuben Grech, Neville Calleja, Paul Bezzina

**Affiliations:** 1 Faculty of Health Sciences, University of Malta, Msida, Malta; 2 Faculty of Medicine & Surgery, University of Malta, Msida, Malta

**Keywords:** Interventional neuroradiology, Air kerma-area product, Reference air kerma, Fluoroscopy time, Diagnostic reference levels

## Abstract

**Objectives:**

To review the literature on diagnostic reference levels (DRLs) in interventional neuroradiology (INR), summarise reported dose values, and examine the methodologies used for their establishment.

**Materials and methods:**

A scoping review was conducted using SCOPUS, Web of Science, PubMed, and ProQuest. Studies reporting DRLs for INR diagnostic procedures (cerebral angiography, (CA)) and therapeutic procedures (stroke thrombectomy, (ST); aneurysm coiling, (AC); arteriovenous malformation/fistula (AVM/AVF) embolisation) were included. Extracted data comprised dose metrics, sample size, percentile definition, procedure classification, and statistical approaches used for DRL derivation.

**Results:**

Thirty-nine studies reported DRLs for air kerma–area product (P_KA_), fluoroscopy time (FT), and reference air kerma (RAK). Most studies defined DRLs using the 75th percentile, although variations were observed in percentile selection, procedure grouping, and inclusion criteria. Considerable heterogeneity in sample sizes and data collection methods was identified. Reported DRLs varied widely: for CA, P_KA_ 41–256.65 Gycm², FT 6–20 min, and RAK 289–921 mGy; for ST, P_KA_ 110–225.1 Gycm², FT 30–45 min, and RAK 730–1590 mGy; for AC, P_KA_ 52.1–487.4 Gycm², FT 16–90 min, and RAK 505–4750 mGy; and for AVM/AVF embolisation, P_KA_ 206.4–550 Gycm², FT 59–135 min, and RAK 2350–6000 mGy.

**Conclusion:**

DRLs in INR show substantial variability, partly driven by methodological inconsistencies. Greater standardisation of DRL derivation and reporting is needed to support harmonisation and optimisation.

**Key Points:**

***Question*** *How does the lack of international consensus on interventional neuroradiology (INR) diagnostic reference levels (DRLs), alongside inconsistent reporting, hinder benchmarking, optimisation, and radiation protection?*

***Findings**** DRLs are reported for major INR procedures, but vary widely across studies and procedure types*.

***Clinical relevance**** Differences in dose metrics, procedure classification, and data collection hinder comparison and benchmarking between centres. Standardised methods and harmonised reporting are crucial for effective dose optimisation and radiation protection in INR. Consistency in deriving DRLs would enable reliable benchmarking and support future registry-based initiatives*.

**Graphical Abstract:**

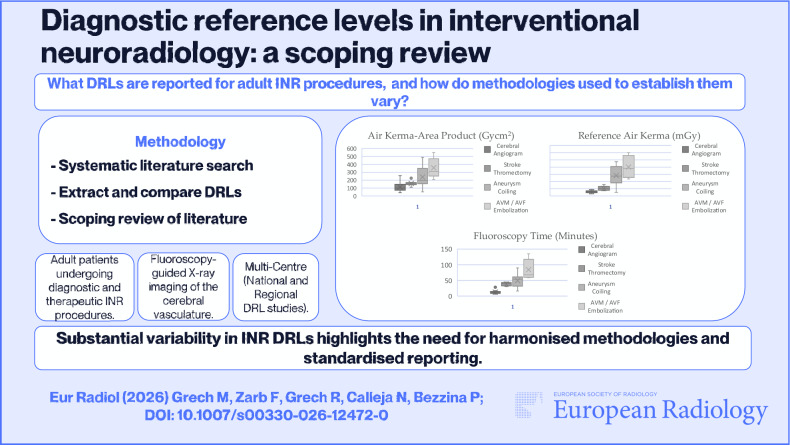

## Introduction

Interventional radiology (IR) has grown over the last decade, with the increase in average life expectancy and technological development, and it often represents a less invasive procedure alternative to surgery [1–4]. The number of IR procedures has increased in the last decade, and these results included only procedures involving imaging tests performed during IR in this period [5], with the actual number of procedures performed now expected to be larger.

When compared to other diagnostic radiographic examinations, IR examinations require higher radiation doses and longer exposure times. When one considers IR’s increased use and high radiation dose, radiation protection and patient dose management are crucial, and efforts to optimise the IR procedures and establishment of diagnostic reference levels (DRLs) during IR procedures are therefore needed [5].

DRLs are a method of investigation of dose levels utilised as a tool to support and monitor optimisation of radiation protection in the medical exposure of patients for diagnostic and interventional procedures [6, 7]. The International Commission on Radiological Protection (ICRP) recommended that DRLs be set at the 75th percentile of the distribution of median values for DRL quantities for specific examinations at individual facilities across a country [6].

New IR systems have dose monitoring devices that track total air kerma-area product (P_KA_), reference air kerma (RAK) at the interventional reference point, fluoroscopy time (FT), and total number of images (NI) recorded during digital acquisition, digital subtraction angiography, or cine runs [7]. All these quantities are considered as appropriate primary or secondary diagnostic reference quantities for IR procedures [8].

Variations exist in the way national DRLs are established, as not all countries may be using the same parameters to define their DRLs. The majority have national DRLs based only on P_KA_, whereas the minority have all three quantities. This limits the possibilities for a thorough comparison due to limited DRL harmonisation [9].

The purpose of this scoping review was to explore the literature for established DRLs in adult interventional neuroradiology (INR) procedures, including both diagnostic and therapeutic applications. The review aims to identify any variations in DRL values, parameters used and evaluate the methodologies applied in their establishment.

The scientific value of this work lies in providing the first comprehensive scoping review dedicated exclusively to DRLs in INR. While individual studies and national reports exist, no prior review has systematically mapped the global literature, synthesised DRL data across the main INR procedures, and examined methodological variations in DRL establishment.

DRLs are typically established at the national level, reflecting local clinical practices, patient demographics, and equipment configurations. While such national DRLs are essential for internal benchmarking, direct international comparisons may be of limited relevance unless methodologies, including procedural definitions, dose indices, and data collection protocols, are standardised.

In addition to mapping existing DRLs, this review undertakes a critical synthesis of the methodological approaches used in their establishment. Given the substantial heterogeneity observed across studies, the review also aims to provide concrete, evidence-informed recommendations to support future harmonisation of DRLs in INR at national and international levels.

## Materials and methods

Ethical approval was not required since this was a review of published literature.

An extensive electronic literature search on DRLs for INR diagnostic procedures (cerebral angiography) and therapeutic procedures (stroke thrombectomy, aneurysm coiling, arteriovenous malformations (AVM) and/or arteriovenous fistulas (AVF) embolisations) was carried out using databases such as ‘Scopus’, ‘Web of Science’, ‘PubMed’, and ‘ProQuest’ for any publications between 1998 to 2023. To enhance the sensitivity of the search, free text and subject headings (MeSH) were utilised. Five main keywords used for the search were: ‘interventional neuroradiology’, ‘air kerma-area product’, ‘reference air kerma’, ‘fluoroscopy time’, and ‘DRLs’. Boolean combinations are provided as Supplementary Material Table 1.

**Fig. 1:**
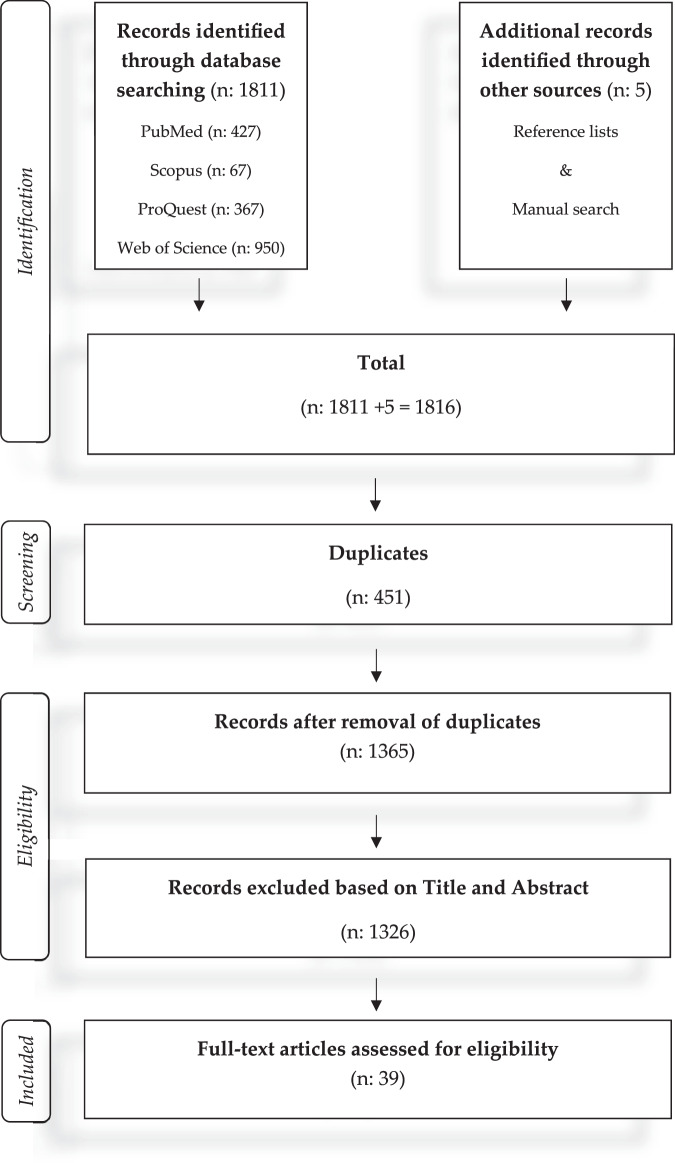
Systematic literature search flowchart

Supplementary Material Table 2 presents the eligibility criteria for selected papers. Eligible publications were classified according to their major topic, which drove the formation of sections and subsections within this literature review. Figure 1 presents the flow of the systematic literature search, which ended up with a total of 39 papers being identified during the period 1998 to 2023.

Two independent reviewers (the researcher, an interventional radiographer with 27 years of experience, and a neuro-interventional radiologist with 25 years of experience) screened titles and abstracts against predefined inclusion and exclusion criteria (Supplementary Material Table 2). Full-text articles were then assessed for eligibility. Discrepancies between reviewers were resolved through discussion, with a third reviewer (an academic in radiography with more than 30 years of experience and with a special interest in interventional radiography) consulted when consensus could not be reached.

A scoping review methodology was chosen over a systematic review or meta-analysis due to the heterogeneity of the available literature. Studies varied widely in procedural classifications, dose indices reported, patient populations, and data collection methods, precluding the application of uniform inclusion criteria necessary for quantitative synthesis. The scoping review approach provided the flexibility to capture the breadth of available evidence, summarise DRL values across diverse contexts, and identify methodological gaps, thereby laying the groundwork for future systematic reviews when greater standardisation in reporting has been achieved.

Data from eligible studies were systematically extracted into a pre-designed spreadsheet capturing: study design, country, patient population, procedure type, dose indices reported (P_KA_, RAK, FT) and methodological details such as collection techniques and equipment used.

DRL comparisons between studies were performed using the Kruskal–Wallis test to determine whether there is a statistically significant difference between the medians of P_KA_, FT, and RAK [10]. The post hoc Dunn’s test was conducted when the results of the Kruskal–Wallis tests were statistically significant, and this test determined exactly which groups are different. All statistical tests were carried out at a significance level (α) of 0.05.

## Results

### Characteristics of included DRL studies

From the 39 studies selected, 22 were prospective and 17 were retrospective studies, with all studies conducted using patient participants. Only one study also reported the use of phantoms.

### Recorded DRLs as per literature findings

The procedures for which DRLs were established in the literature were: cerebral angiography, stroke thrombectomy, aneurysm coiling, and AVM/AVF embolisation.

### Cerebral angiography

Table 1 shows the DRLs (3rd quartile values) for P_KA_, FT, and RAK for all diagnostic cerebral angiography procedures, with those studies having their DRLs above the weighted mean shaded grey.

**Table 1:** Cerebral angiography DRLs (3rd quartile values)

Author	Year	Country	Institutions	Sample size	P_KA_ (DRL)	FT (DRL)	RAK (DRL)
			(*N*)	(*N*)	(Gy/cm^2^)	(minutes)	(mGy)
McParland et al [44]	1998	Saudi Arabia	1	28	82.5		
Brambilla et al [45]	2004	Italy		188	**198**	**17.5**	
Verdun et al [25]	2005	Switzerland	5	91	**124**		
Aroua et al [36]	2007	Switzerland	5	91	**125**	**15**	
Bleeser et al [29]	2008	Belgium	14	616	71		
Vano et al [46]	2008	Europe	3	72	**107**	**12**	
Trueb et al [26]	2009	Switzerland	20	32	**160**	8	
Alexander et al [34]	2010	USA	1		**102.4**		
Sandborg et al [47]	2010	Sweden	1	226	72		
Kien et al [48]	2011	France	9		**229**	**14**	
D’Ercole et al [22]	2012	Italy	1	100	**180.4**	**12.3**	
Zotova et al [11]	2012	Bulgaria	5	67	41	**12.2**	379
Soderman et al [23]	2013	Sweden	1	174	76	9	
Chun et al [38]	2014	South Korea	1	439	**154.2**	**14**	
Erskine et al [37]	2014	Australia	1	257	82.6	6.25	
Ihn et al [16]	2016	South Korea	23	490	**144.2**	**12.2**	**921.1**
Etard et al [27]	2017	France	34	695	87.5	10.3	628
Hassan et al [17]	2017	France	1	398	59.7	7.5	450
Sailer et al [49]	2017	Netherlands	1	112	44.5		
Acton et al [28]	2018	Ireland	1	189	96		
Rana et al [33]	2018	India	1	226	**121.97**		
Choi et al [35]	2019	South Korea	1	540	94.1	**13.8**	**690**
Rizk et al [13]	2019	Lebanon	6	310	83	6	**693**
Malan et al [15]	2020	South Africa	1	61	55	**14**	289
Ihn et al [18]	2021	South Korea	22	429	101.6	**13.3**	**711.3**
Papanastasiou et al [30]	2021	Greece	1	60	70.2	9.2	494
Opitz et al [12]	2022	Germany	1	71	**256.65**	**17.18**	
Tristram et al [9]	2022	Germany	1	314	100.6	**15.3**	525
Slave et al [14]	2023	South Africa	1	26	**209.3**	**28.4**	**868.5**
				Weighted mean	102.64	11.73	654.74

Bold values above the weighted mean

The lowest P_KA_ reported was 41 Gycm^2^ [11] while the highest level was 256.65 Gycm^2^ [12]. The ranges for FT varied from a minimum of 6 min [13] to a maximum of 28 min [14]. The minimum RAK was 289 mGy [15] and the maximum was 921.1 mGy [16].

### Stroke thrombectomy

Table 2 shows the DRLs (3rd quartile values) for P_KA_, FT, and RAK for all stroke thrombectomy procedures, with those studies having their DRLs above the weighted mean shaded grey.

**Table 2:** Stroke thrombectomy 3rd quartile values

Author	Year	Country	Institutions	Sample size	P_KA_ (DRL)	FT (DRL)	RAK (DRL)
			(*N*)	(*N*)	(Gy/cm^2^)	(minutes)	(mGy)
Hassan et al [17]	2017	France	1	73	110	30	1018
Acton et al [28]	2018	Ireland	1	10	**172**		
Farah et al [32]	2018	France	1	319	162	**42**	854
Guenego et al [19]	2019	International	5	520	148		730
Schegerer et al [31]	2019	Germany			158	35	
Pace et al [50]	2020	Malta	1	122	144.7		
Ihn et al [18]	2021	South Korea	22	326	**225.1**	**44.7**	**1590**
Tristram et al [9]	2022	Germany	1	457	151.9	40.3	**1032**
				Weighted mean	163.57	41.34	1012.57

Bold values above the weighted mean

The P_KA_ range was from a minimum of 110 Gycm^2^ [17] to a maximum of 225.1 Gycm^2^ [18]. The lowest FT range was 30 min [17], while the highest was almost 45 min [18]. The DRL in RAK ranged from 730 mGy [19] to 1590 mGy [18].

### Aneurysm coiling

Table 3 presents the DRLs (3rd quartile values) from 26 studies for P_KA_, FT, and RAK, for all aneurysm coiling procedures, with those studies having their DRLs above the weighted mean shaded grey. One study by Forbrig et al [20] was not included in the comparative DRL Table 3, as aneurysm coiling procedures were stratified into four distinct categories. This categorisation limited the ability to directly compare their reported DRLs with those from other studies, which typically present aggregated data for aneurysm coiling as a single category. The lowest P_KA_ was 52.1 Gycm^2^ [21], whereas the highest was 487.4 Gycm^2^ [22]. The lowest FT reported was that of 16 min [23] while the highest was 90 min [24]. The highest RAK was 4750 mGy [24], while the lowest was 505 mGy [15].

**Table 3:** Aneurysm coiling 3rd quartile values

Author	Year	Country	Institutions	Sample size	P_KA_ (DRL)	FT (DRL)	RAK (DRL)
			(*N*)	(*N*)	(Gy/cm^2^)	(minutes)	(mGy)
Brambilla et al [45]	2004	Italy					**4240**
Verdun et al [25]	2005	Switzerland	5	58	**352**	50	
Aroua et al [36]	2007	Switzerland	5	58	**440**	50	
Miller et al [24]	2009	USA	7	148	**339.4**	**90**	**4750**
Vano et al [51]	2009	Spain	1	325	**386**		**3900**
Alexander et al [34]	2010	USA	1		167.3		
Sandborg et al [47]	2010	Sweden	1	226	157		
Vano et al [51]	2010	Spain	1	383	**392**		3300
Kien et al [48]	2011	France	9		**349**	**58**	
D’Ercole et al [22]	2012	Italy	1	72	**487.4**	46.3	
Soderman et al [23]	2013	Sweden	1	138	196	16	
Chun et al [38]	2014	South Korea	1	111	**272.8**	**61.1**	
Erskine et al [37]	2014	Australia	1	91	152.9	32	
Borota et al [52]	2016	Sweden	1	35	97.39	**83.05**	1980
Ihn et al [16]	2016	South Korea	23	371	**271**	**64.7**	**4471.3**
Etard et al [27]	2017	France	19	427	186.5	**58**	2763
Hassan et al [17]	2017	France	1	71	111.9	34.8	1368
Acton et al [28]	2018	Ireland	1	109	123		
Rana et al [33]	2018	India	1	54	**370.78**		
Choi et al [35]	2019	South Korea	1	173	206.2	**60**	
Rizk et al [13]	2019	Lebanon	3	117	190	27	2422
Schegerer et al [31]	2019	Germany			192	54	
Malan et al [15]	2020	South Africa	1	55	63	25	505
Peter et al [21]	2020	South Africa	1	30	52.1	17.8	
Ihn et al [18]	2021	South Korea	22	327	199.9	**57.3**	**3458.7**
Tristram et al [9]	2022	Germany	1	129	186.8	**70**	1906
Slave et al [14]	2023	South Africa	1	15	**275**	34.1	1744
				Weighted mean	254.06	54.51	3309.89

Bold values above the weighted mean

### AVM/AVF embolisation

As per previous procedures, Table 4, shows the DRLs (3rd quartile values) for P_KA_, FT, and RAK for all AVM/AVF embolisation procedures with those studies having their DRLs above the weighted mean shaded grey.

**Table 4:** AVM/AVF embolisation 3rd quartile values

Author	Year	Country	Institutions	Sample size	P_KA_ (DRL)	FT (DRL)	RAK (DRL)
			(*N*)	(*N*)	(Gy/cm^2^)	(minutes)	(mGy)
Miller et al [24]	2009	USA	7	134	**550**	**135**	**6000**
Sandborg et al [47]	2010	Sweden	1	226	225		
Kien et al [48]	2011	France	9		**435**	61	
Etard et al [27]	2017	France	13	239	280.5	67.8	3224
Hassan et al [17]	2017	France	1	33	206.4		2350
Acton et al [28]	2018	Ireland	1	6	310		
Rana et al [33]	2018	India	1	7	288.54		
Ihn et al [18]	2021	South Korea	22	78	**412.3**	**99.3**	**4447.8**
Opitz et al [12]	2022	Germany	1	111	**507.33**	58.57	
				Weighted mean	348.63	86.37	4130.20

Bold values above the weighted mean

The lowest P_KA_ was 206.4 Gy/cm^2^ [17] and the highest 550 Gy/cm^2^ [24]. The lowest FT was approximately 59 min [12], while the highest was 135 min [24]. The RAK range was 2350 mGy [17] to 6000 mGy [24].

## Discussion

Although formal quality assessment was not conducted, a qualitative appraisal of the included sources revealed considerable variation in methodological transparency, sample sizes, and national endorsement. DRLs derived from national surveys or endorsed by regulatory/professional bodies were considered more reliable and broadly applicable. Conversely, single-centre studies or those lacking detail on dose index definitions were less robust. The completeness of reported dose indices also varied, with some sources reporting only P_KA_, while others included RAK and FT, limiting cross-study comparability.

Variations in patient doses were revealed in published studies between the contributing hospitals and at times even the same hospital for the same procedure [12, 13, 15, 25–28]. These variations in patient doses were attributed to the procedure complexity, patient morphology, type of equipment, imaging techniques and protocols, different dose reduction technologies, operators’ skills and experience, and standardised method of data collection [12, 13, 15, 25–28].

As DRLs are generally defined at the national level, variations in methodology limit the direct comparability of values between countries. Without standardised approaches to parameter selection, data collection, and analysis, observed differences may reflect methodological divergence rather than genuine practice variation.

### Procedure complexity

**Fig. 2:**
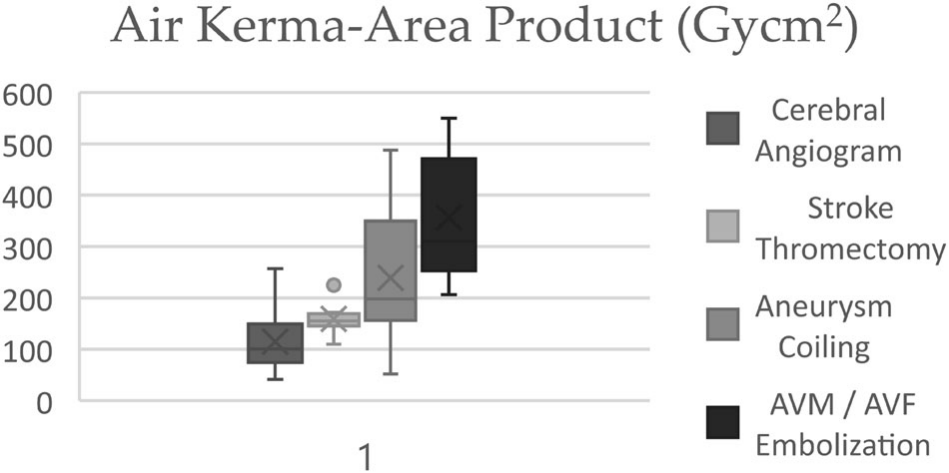
Box-and-whisker plot showing P_KA_ (Gycm^2^) variations amongst the procedures (*p* < 0.001)

**Fig. 3:**
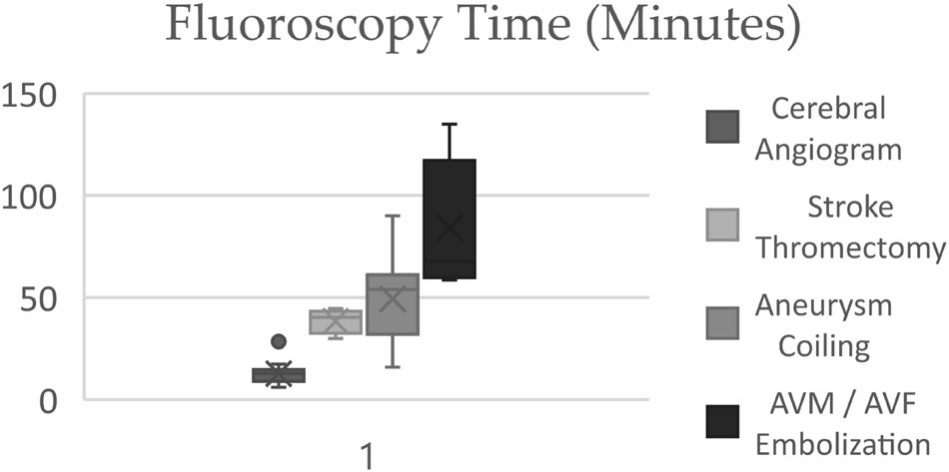
Box-and-whisker plot showing FT (minutes) variations amongst the procedures (*p* < 0.001)

**Fig. 4:**
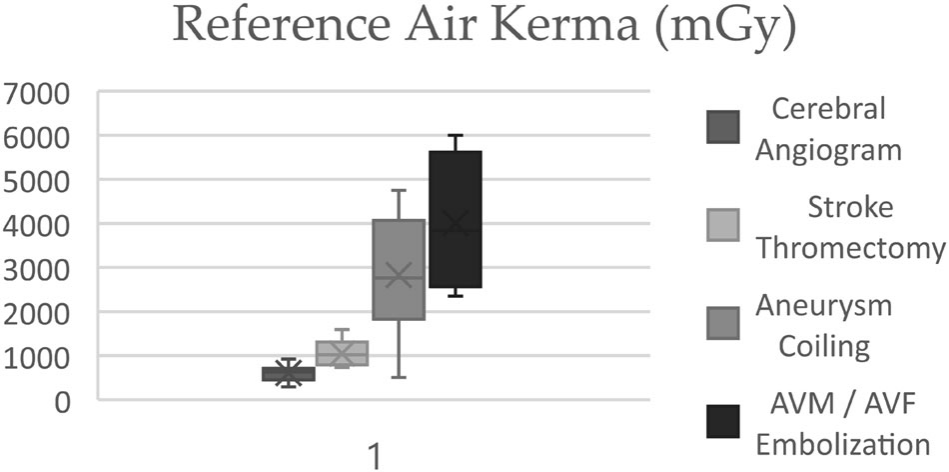
Box-and-whisker plot showing RAK (mGy) variations amongst the procedures (*p* < 0.001)

The complexity of an interventional procedure greatly impacts the amount of radiation administered [2]. Ideally, interventional procedures should have DRLs based on their difficulty level as suggested by ICRP 2017 [27]. Several reviewed studies have proposed local DRLs based on complexity for INR procedures, such as cerebral angiography, intracranial aneurysm embolisation, and arteriovenous malformations [22, 29–31]. The complexity demonstrates the ability to identify factors for INR procedures, categorising them as easy, medium, or complicated, and assigning DRL values accordingly [6]. Quantifying procedure complexity requires extensive clinical data, which is not always available, especially in retrospective research [27]. However, dividing the patients into complexity categories has further reduced the number of patients in each category, resulting in weakening the strength of the corresponding comparisons [30]. It may be the case that more complex procedures would require a longer FT, which may result in a higher P_KA._ The rating of complexity for this review was worked out based on the dose administered in P_KA,_ FT, and RAK. Detailed P_KA,_ FT and RAK for each procedure type from different authors are illustrated in Figs. 2–4.

The Kruskal–Wallis test indicated that for P_KA_, FT and RAK (Figs. 2–4), there is a significant difference in the P_KA_, FT and RAK between the different groups (*p* < 0.001). Since the results were statistically significant, post hoc Dunn’s test using a Bonferroni corrected alpha of 0.0083 was conducted. This test indicated that the mean ranks of P_KA_, FT and RAK for the following pairs are significantly different between them (*p* < 0.05).

Aneurysm coiling (45.23 Gycm^2^) gives almost double the dose, and AVM/AVF embolisation (61.67 Gycm^2^) gives almost triple the dose in terms of P_KA_ when compared to cerebral angiography (21.45 Gycm^2^). This was also noted when AVM/AVF embolisation gave almost double the dose when compared to stroke thrombectomy (34.38 Gycm^2^). From these results, AVM/AVF embolisation is indicated as the most complex procedure.

Cerebral angiography FT (11.29 min) was 3 times lower when compared to FT in aneurysm coiling (35.3 min) and 4 times lower when compared to the AVM/AVF embolisation (46.2 min). This shows that the more FT was required, the more complex the procedure.

When comparing RAK data of cerebral angiography (7 mGy) with aneurysm coiling (14.4 mGy) and AVM/AVF embolisation (27.5 mGy), this was almost double and triple that of the diagnostic procedure. From the mentioned results, aneurysm coiling and AVM/AVF embolisations have higher radiation doses due to being more complicated procedures.

Based on the indications from the findings of K_PA_, FT, and RAK measures in the reviewed literature, a rate from 1 to 4 was assigned according to the procedure complexity, where 1 indicates the least complex and 4 being the most complex: cerebral angiography—1; stroke/mechanical thrombectomy—2; aneurysm coiling—3, and AVM/AVF embolisation—4.

### Patient morphology

The difference in dose is influenced by several variables, including but not limited to patient factors, lesions, or pathological factors [14].

Adult DRLs are frequently specified for people of average size [28]. However, patients’ gender did not influence the radiation dose to the patients during cerebral angiography. Studies did not consider the impact of patient morphology on the dose because the process takes place at the level of the head, which is not affected by gender [24, 32]. Nevertheless, collecting patient’s body mass index, which may be a better predictor of dose than gender, was still important for neuro-interventions, as the initial access route may be from the femoral artery moving the catheter across the abdominal and thoracic areas, where the patient’s anatomy and thickness may result in higher doses in men compared to women [32].

When compared to anterior circulation aneurysms, posterior cerebral circulation aneurysms are more difficult to treat due to unfavourable anatomy, a more frequent requirement for assist devices such as embolisation devices, balloon remodelling, and stents, which have a higher complication risk [28]. Separate DRLs were proposed for anterior and posterior circulation coiling procedures since aneurysm location is the main factor affecting radiation exposure during coiling procedures; however, this evaluation could not be performed from the reviewed studies evaluated as the location of the coils was not indicated [28].

### Type of equipment

Higher P_KA_ may result from older equipment due to image intensifier deterioration becoming less sensitive to x-ray photons. To keep the same image quality, radiation exposures with older intensifiers must be increased [13, 23, 33]. The detectors used for IR have advanced over time [13, 33]. Flat panel detectors and pulsed fluoroscopy are now standard features on IR equipment [13, 23, 33].

Over the years, results demonstrated a gradual decrease in reported P_KA_, owing to the implementation of digital technology, continuous optimisation of hardware and software for data acquisition and image generation, advancements in radiation protection systems integrated into x-ray equipment, improvement in fluoroscopy image quality and the possibility of storing the last image of a fluoroscopy series reducing the number of acquisitions. All these factors could explain radiation dose reductions [13, 23, 27, 33, 34].

Two types of equipment were encountered in the literature: conventional, that is, the Image Intensifier, and digital, a Flat Panel detector. The radiation dose provided by these two categories of equipment was compared using the Mann–Whitney test.

**Fig. 5:**
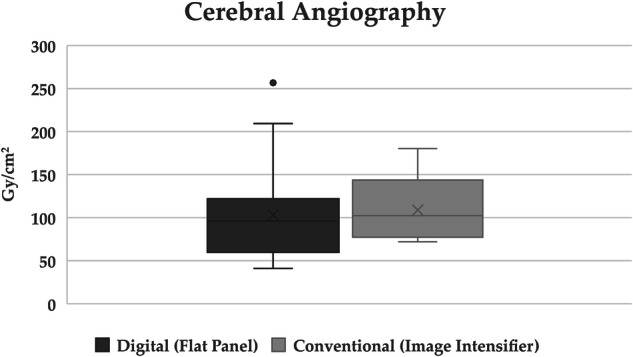
Comparison of median values of P_KA_ (Gy/cm^2^) in cerebral angiography (*p* = 0.57)

**Fig. 6:**
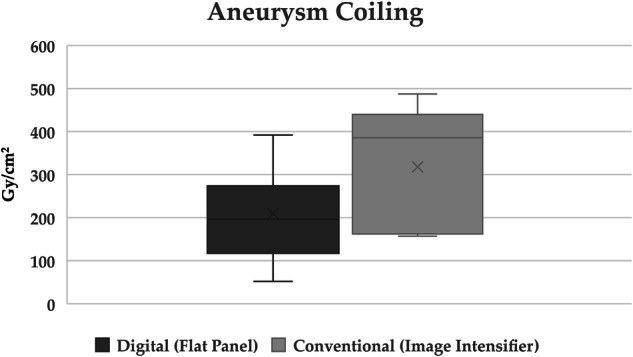
Comparison of median values of P_KA_ (Gy/cm^2^) in aneurysm coiling (*p* = 0.20)

Differences of the median in the two types of equipment in both cerebral angiography (*n* = 24) and aneurysm coiling (*n* = 22) did not achieve statistical significance (*p* > 0.05). This could have been due to the small population sample size, as it may have been too small to demonstrate any significant variation in the values (Figs. 5 and 6).

Variations in radiation dose indicated that, although not statistically significant, with the use of modern digital equipment, less radiation dose was administered when compared to conventional equipment.

### Imaging techniques and protocols

Variations in DRLs can be attributed to a variety of reasons, including differences in imaging techniques and protocols used between centres [22]. A considerable dose variance is produced by differences in the number of frames, projections, and exposure parameters utilised in the procedures [22].

Interventional neuroradiologists attempt to use low pulse rate fluoroscopy whenever possible to reduce radiation exposure for the patient. The use of high FT values in some procedures may be explained using higher fluoroscopic rates [9, 11].

Different work practices may have an impact on the administered dose. Automated exposure algorithms, determined to be the most significant factor in influencing patient dose, may be used to manage fluoroscopy interventional procedures [16, 18].

### Different dose reduction technologies

Roadmap fluoroscopy is one advanced technological method that allows for the visualisation of anatomic structures using image subtraction at peak intravenous contrast opacification, resulting in a significant reduction in the amount of image frames enabling dose savings [35]. Lower frame rates that give acceptable image quality could be used to reduce the radiation exposure by reducing the number of frames [35].

### Neuro-interventional radiologists skills and experience

Significant variation was demonstrated in P_KA_, FT, and the number of images obtained during cerebral angiography procedures conducted by experienced radiologists [36]. Lack of expertise among young radiology trainees is a primary cause of dose variability [36]. First-year training fellows are more likely to utilise more FT during the procedure than experienced operators and ascribed significant dose increases in teaching hospitals [36].

### Standardised method of data collection

Since there is no broad agreement on adequate dosimetric quantities for radiation dose estimation, and because multiple dose metrics are used in published studies, comparing radiation doses at different sites becomes challenging [13, 23, 33]. Due to the lack of a standardised technique for collecting and reporting radiation exposure during neuro-interventional treatments, it was suggested that, to be able to compare practices in accordance with radiation safety rules, a multicentric database with standardised data as well as uniform neuro-interventional nomenclature is required [17].

### Methodological synthesis and implications for DRL harmonisation

A critical examination of the included studies highlighted substantial variability in how DRLs were defined, calculated, and reported. Differences were observed in the selection of dose indices (e.g., P_KA_ alone versus full reporting of P_KA_, RAK, and FT), inconsistencies in procedural categorisation (such as grouping initial and follow-up angiography together or combining coiling with diagnostic runs), and a lack of clarity regarding whether CBCT and ancillary acquisitions were included in DRL calculations. These methodological inconsistencies complicate interpretation and limit the reliability of cross-study comparisons, as reported differences may reflect variations in reporting practices rather than true differences in clinical performance or patient risk.

Furthermore, the heterogeneity in data collection approaches, ranging from manual extraction to automated system logs without standardised definitions, underscores the absence of a unified methodological framework. The lack of consistent reporting of patient morphology, equipment characteristics, fluoroscopic frame rates, and procedural complexity further restricts the ability to stratify and benchmark DRLs meaningfully. These findings emphasise the need for greater methodological alignment across institutions and countries to ensure that DRLs can fulfil their intended role as optimisation and benchmarking tools.

To support such harmonisation, several methodological priorities emerge from this synthesis. These include the adoption of a core set of dose indices, agreement on standardised procedural taxonomies, consistent inclusion or exclusion rules for CBCT and additional imaging runs, mandatory reporting of complexity and key patient and equipment factors, and the development of standardised data dictionaries and collection templates. Together, these measures would enhance DRL comparability, improve transparency, and enable robust regional or international analyses.

High P_KA_ is usually associated with high RAK [14]. The link between P_KA_ and FT was weaker, which was similar to earlier findings [30, 37]. No single dose parameter may be extrapolated solely to infer radiation exposure; instead, during radiation exposure optimisation, as many parameters as possible should be considered. This is supported by the ICRP’s recommendations [6].

The following examples from the literature reviewed listed below confirm the lack of standardisation in data collection methods for DRL establishment.

Initial diagnostic angiography and follow-up angiographies were not separated as different categories [17]. While initial diagnostic angiography generally refers to the examination of four or more cerebral vessels, follow-up angiography will normally study two or three arteries, with the exception of AVM/AVF, resulting in a reduced radiation dose, and so their DRLs should be separate [17].

When diagnostic cerebral angiographies and endovascular coil embolisations were conducted in the same session, patient data were separated into two groups [38]. To date, this was the only published report with such segregated data [38]. Previous studies may have included these procedures under the coiling embolisation group, which may have increased the reported DRL for embolisation procedures [22, 24, 25, 36]. This was also reported by Papanastasiou et al [30], who explained that since comparable data were not included as independent entries in the system dose reports, their study did not provide a separate analysis of the doses delivered to patients by cone beam CT (CBCT) acquisitions conducted as part of the procedure. CBCT is only conducted during cerebral Angiograms, and CBCT dose data is put into system dose reports as part of the procedure [30].

This highlights the importance of a harmonised way of categorising interventional procedures. If the same procedure varies in the way it is conducted, then DRLs cannot be compared.

Although published after the predefined inclusion period, the recent study by Lopes et al [39] contributes significantly to the evolving landscape of DRLs in INR. This multicentre European study provides updated and procedure-specific DRLs for key INR procedures, including mechanical thrombectomy and aneurysm coiling, based on a large and diverse dataset. The findings not only offer contemporary benchmarks for radiation exposure but also highlight variation in practices across centres, reinforcing the need for harmonised methodologies in DRL establishment. Furthermore, the study exemplifies the importance of coordinated data collection and reporting standards at the European level, which may serve as a model for future regional or global DRL development initiatives.

Continental comparisons (e.g., between Europe (*n* = 26), Asia (*n* = 7), the Americas (*n* = 2), Australia (*n* = 1), and Africa (*n* = 3)) were not feasible due to the predominance of European studies and the limited number of comparable studies from other regions, highlighting a gap in globally representative DRL data.

While there is no unified international guideline specific to INR, relevant recommendations from ICRP [6, 40, 41] and European Commission documents guide radiation protection practices [42, 43], including the use of DRLs. Several national bodies have developed procedure-specific DRLs for INR, but these vary significantly in methodology and scope, underscoring the need for harmonised international guidance.

This scoping review demonstrates that, although DRLs have been reported for several INR procedures, including CA, ST, AC, and AVM/AVF embolisation, substantial variability persists in both reported dose values and underlying methodologies. DRL coverage remains limited or inconsistent for less frequently performed procedures, such as tumour embolisation, as well as for paediatric neuro-interventions. Furthermore, only a minority of studies consistently report all three key dose indicators (PKA, RAK, and FT), which significantly hampers standardisation and meaningful cross-country comparison.

These findings highlight an urgent need for methodological harmonisation in future DRL development for INR. Central to this effort is the adoption of a harmonised minimum dataset that mandates the routine reporting of PKA, RAK, and FT as core dose indices, alongside standardised procedural classifications that clearly distinguish between diagnostic and follow-up angiography, standalone vs combined therapeutic procedures, and the use of CBCT. In parallel, structured reporting of key variables known to influence patient dose, such as patient morphology, procedural complexity, frame rates, and angiography system generation, should be implemented to improve data interpretability and comparability. To support sustainable harmonisation, future work should prioritise multicentre and multinational collaborations and the development of registries underpinned by predefined data dictionaries, enabling consistent data collection and facilitating European or international DRL initiatives. Finally, regular review and updating of DRLs are essential to ensure alignment with ongoing advances in technology, evolving interventional techniques, and optimisation strategies in neuro-interventional practice.

## Conclusion

This work is original in its scope, covering both diagnostic and therapeutic INR procedures and including studies from multiple continents over a 25-year period. It presents DRL distributions, highlighting variability and outliers not previously consolidated in this field. It also offers methodological insights by identifying key differences in dose indices, procedural categorisation, and data collection approaches, all of which affect international harmonisation. By mapping these gaps and variations, the review provides a reference framework to support future DRL development, standardisation, and policy making in INR radiation protection.

## Supplementary information

ELECTRONIC SUPPLEMENTARY MATERIAL

## Abbreviations

AC: Aneurysm coiling
AVF: Arteriovenous fistulas
AVM: Arteriovenous malformations
CA: Cerebral angiography
CBCT: Cone beam computerised tomography
DRLs: Diagnostic reference levels
FT: Fluoroscopy time
Gycm^2^: Gray-centimetres squared
ICRP: International Commission on Radiological Protection
INR: Interventional neuroradiology
IR: Interventional radiology
mGy: Milligray
min: Minutes
P_KA_: Air kerma area product
RAK: Reference air kerma
ST: Stroke thrombectomy

## References

[1] UNSCEAR (2010) UNSCEAR 2008 report, vol 2. Sources and effects of ionizing radiation. UNSCEAR, Vienna

[2] International Atomic Energy Agency (IAEA) (2009) Establishing guidance levels in X ray guided medical interventional procedures: a pilot study. Safety Reports Series No. 59. IAEA, Vienna

[3] Vañó E, Rosenstein M, Liniecki J, Rehani MM, Martin CJ, Vetter RJ (2009) ICRP Publication 113. Education and training in radiological protection for diagnostic and interventional procedures. Ann ICRP 39:7–68. 10.1016/j.icrp.2011.01.002 21788173 10.1016/j.icrp.2011.01.002

[4] Nekolla EA, Schegerer AA, Griebel J, Brix G (2017) Häufigkeit und dosis diagnostischer und interventioneller Röntgenanwendungen: trends zwischen 2007 und 2014 [Frequency and doses of diagnostic and interventional X-ray applications: trends between 2007 and 2014]. Radiologe 57:555–562. 10.1007/s00117-017-0242-y 28361179 10.1007/s00117-017-0242-y

[5] Lee MY, Kwon J, Ryu GW, Kim KH, Nam HW, Kim KP (2019) Review of national diagnostic reference levels for interventional procedures. Prog Med Phys 30:75–88. 10.14316/pmp.2019.30.4.75

[6] Vañó E, Miller DL, Martin CJ et al (2017) ICRP Publication 135: Diagnostic reference levels in medical imaging. Ann ICRP 46:1–144. 10.1177/0146645317717209 10.1177/0146645317717209 29065694

[7] (2007) The 2007 recommendations of the International Commission on Radiological Protection. ICRP publication 103. Ann ICRP 37:1–332. 10.1016/j.icrp.2007.10.003 10.1016/j.icrp.2007.10.003 18082557

[8] European Union (2014) Council directive 2013/59/Euratom on laying down basic safety standards for protection against the dangers arising from exposure to ionising radiation, and repealing directives 89/618/Euratom, 90/641/Euratom, 96/29/Euratom, 97/43/Euratom and 2003/122/Euratom. Off J Eur Union L 13:1–73

[9] Tristram J, Steuwe A, Kröpil F et al (2022). Typical doses and typical values for fluoroscopic diagnostic and interventional procedures. J Radiol Prot. 10.1088/1361-6498/ac5294 10.1088/1361-6498/ac5294 35130526

[10] Kruskal WH, Wallis WA (1952) Use of ranks in one-criterion variance analysis. J Am Stat Assoc 47:583–621. 10.1080/01621459.1952.10483441

[11] Zotova R, Vassileva J, Hristova J, Pirinen M, Järvinen H (2012) A national patient dose survey and setting of reference levels for interventional radiology in Bulgaria. Eur Radiol 22:1240–1249. 10.1007/s00330-012-2386-5 22350490 10.1007/s00330-012-2386-5

[12] Opitz M, Zensen S, Bos D et al (2022) Radiation exposure in the endovascular therapy of cranial and spinal dural arteriovenous fistula in the last decade: a retrospective, single-center observational study. Neuroradiology 64:587–595. 10.1007/s00234-021-02816-6 34570252 10.1007/s00234-021-02816-6 PMC8850286

[13] Rizk C, Farah J, Vanhavere F, Fares G (2019) National diagnostic reference levels in interventional radiology suites in Lebanon: a multicenter survey. Radiat Prot Dosim 187:50–60. 10.1093/rpd/ncz137 10.1093/rpd/ncz137 31111937

[14] Slave O, Mahomed N (2023) An audit of patient radiation doses in interventional radiology at a South African hospital. SA J Radiol 27:2559. 10.4102/sajr.v27i1.2559 36756356 10.4102/sajr.v27i1.2559 PMC9900283

[15] Malan L, Pitcher RD, da Silva M, Breuninger S, Groenewald W (2021) Diagnostic reference levels for fluoroscopically guided procedures in a South African tertiary hospital. Acta Radiol 62:807–814. 10.1177/0284185120938371 32640888 10.1177/0284185120938371

[16] Ihn YK, Kim BS, Byun JS et al (2016) Patient radiation exposure during diagnostic and therapeutic procedures for intracranial aneurysms: a multicenter study. Neurointervention 11:78–85. 10.5469/neuroint.2016.11.2.78 27621943 10.5469/neuroint.2016.11.2.78 PMC5018552

[17] Hassan AE, Amelot S (2017) Radiation exposure during neurointerventional procedures in modern biplane angiographic systems: a single-site experience. Interv Neurol 6:105–116. 10.1159/000456622 29118787 10.1159/000456622 PMC5662989

[18] Ihn YK, Kim BS, Jeong HW et al (2021) Monitoring radiation doses during diagnostic and therapeutic neurointerventional procedures: multicenter study for establishment of reference levels. Neurointervention 16:240–251. 10.5469/neuroint.2021.00437 34695909 10.5469/neuroint.2021.00437 PMC8561028

[19] Guenego A, Mosimann PJ, Pereira VM et al (2019) Proposed achievable levels of dose and impact of dose-reduction systems for thrombectomy in acute ischemic stroke: an international, multicentric, retrospective study in 1096 patients. Eur Radiol 29:3506–3515. 10.1007/s00330-019-06062-6 30903333 10.1007/s00330-019-06062-6

[20] Forbrig R, Ozpeynirci Y, Grasser M, Dorn F, Liebig T, Trumm CG (2021) Radiation dose and fluoroscopy time of modern endovascular treatment techniques in patients with saccular unruptured intracranial aneurysms. Eur Radiol 30:4504–4513. 10.1007/s00330-020-06777-x. Erratum in: Eur Radiol. 2021;31:8817. 10.1007/s00330-021-07874-1 10.1007/s00330-020-06777-x PMC8093177 32193640

[21] Peter Y, Speelman A, Daries V (2020) Measurement of the average radiation dose to the local skin and thyroid gland during intracranial aneurysm coil embolization. Radiography 27:255–259. 10.1016/j.radi.2020.07.019 32807613 10.1016/j.radi.2020.07.019

[22] D’Ercole L, Thyrion FZ, Bocchiola M, Mantovani L, Klersy C (2012) Proposed local diagnostic reference levels in angiography and interventional neuroradiology and a preliminary analysis according to the complexity of the procedures. Phys Med 28:61–70. 10.1016/j.ejmp.2010.10.008 21074469 10.1016/j.ejmp.2010.10.008

[23] Söderman M, Mauti M, Boon S et al (2013) Radiation dose in neuroangiography using image noise reduction technology: a population study based on 614 patients. Neuroradiology 55:1365–1372. 10.1007/s00234-013-1276-0 24005833 10.1007/s00234-013-1276-0 PMC3825538

[24] Miller DL, Kwon D, Bonavia GH (2009) Reference levels for patient radiation doses in interventional radiology: proposed initial values for U.S. practice. Radiology 253:753–764. 10.1148/radiol.2533090354 19789226 10.1148/radiol.2533090354 PMC2786193

[25] Verdun FR, Aroua A, Trueb PR, Vock P, Valley JF (2005) Diagnostic and interventional radiology: a strategy to introduce reference dose level taking into account the national practice. Radiat Prot Dosim 114:188–191. 10.1093/rpd/nch547 10.1093/rpd/nch547 15933106

[26] Trueb PR, Aroua A, Stuessi A et al (2009) Diagnostic reference levels in cardiology and interventional radiology. In: Dössel O, Schlegel WC (eds) IFMBE Proceedings of the world congress on medical physics and biomedical engineering, 7–12 Sept 2009, Munich, vol 25/3. Springer, Berlin. 10.1007/978-3-642-03902-7_39

[27] Etard C, Bigand E, Salvat C et al (2017) Patient dose in interventional radiology: a multicentre study of the most frequent procedures in France. Eur Radiol 27:4281–4290. 10.1007/s00330-017-4780-5 28289939 10.1007/s00330-017-4780-5

[28] Acton H, James K, Kavanagh RG et al (2018) Monitoring neurointerventional radiation doses using dose-tracking software: implications for the establishment of local diagnostic reference levels. Eur Radiol 28:3669–3675. 10.1007/s00330-018-5405-3 29651772 10.1007/s00330-018-5405-3

[29] Bleeser F, Hoornaert MT, Smans K et al (2008) Diagnostic reference levels in angiography and interventional radiology: a Belgian multi-centre study. Radiat Prot Dosim 129:50–55. 10.1093/rpd/ncn028 10.1093/rpd/ncn028 18283058

[30] Papanastasiou E, Protopsaltis A, Finitsis S, Hatzidakis A, Prassopoulos P, Siountas A (2021) Institutional diagnostic reference levels and peak skin doses in selected diagnostic and therapeutic interventional radiology procedures. Phys Med 89:63–71. 10.1016/j.ejmp.2021.07.029 34352677 10.1016/j.ejmp.2021.07.029

[31] Schegerer A, Loose R, Heuser LJ, Brix G (2019) Diagnostic reference levels for diagnostic and interventional X-ray procedures in Germany: update and handling. Rofo 191:739–751. 10.1055/a-0824-7603 30665250 10.1055/a-0824-7603

[32] Farah J, Rouchaud A, Henry T et al (2019) Dose reference levels and clinical determinants in stroke neuroradiology interventions. Eur Radiol 29:645–653. 10.1007/s00330-018-5593-x 30019142 10.1007/s00330-018-5593-x

[33] Rana BS, Kumar S, Ahuja CK, Singh NP, Yadav MK, Sandhu IS (2018) Estimation of radiation exposure to the patients in diagnostic and therapeutic interventional procedures. Radiat Prot Dosim 181:290–300. 10.1093/rpd/ncy025 10.1093/rpd/ncy025 29462376

[34] Alexander MD, Oliff MC, Olorunsola OG, Brus-Ramer M, Nickoloff EL, Meyers PM (2010) Patient radiation exposure during diagnostic and therapeutic interventional neuroradiology procedures. J Neurointerv Surg 2:6–10. 10.1136/jnis.2009.000802 21990551 10.1136/jnis.2009.000802

[35] Choi J, Kim B, Choi Y et al (2019) Image quality of low-dose cerebral angiography and effectiveness of clinical implementation on diagnostic and neurointerventional procedures for intracranial aneurysms. AJNR Am J Neuroradiol 40:827–833. 10.3174/ajnr.A6029 30948380 10.3174/ajnr.A6029 PMC7053903

[36] Aroua A, Rickli H, Stauffer JC et al (2007) How to set up and apply reference levels in fluoroscopy at a national level. Eur Radiol 17:1621–1633. 10.1007/s00330-006-0463-3 17072616 10.1007/s00330-006-0463-3

[37] Erskine BJ, Brady Z, Marshall EM (2014) Local diagnostic reference levels for angiographic and fluoroscopic procedures: Australian practice. Australas Phys Eng Sci Med 37:75–82. 10.1007/s13246-014-0244-2 24430258 10.1007/s13246-014-0244-2

[38] Chun CW, Kim BS, Lee CH, Ihn YK, Shin YS (2014) Patient radiation dose in diagnostic and interventional procedures for intracranial aneurysms: experience at a single center. Korean J Radiol 15:844–849. 10.3348/kjr.2014.15.6.844 25469098 10.3348/kjr.2014.15.6.844 PMC4248642

[39] Lopes R, Teles P, Santos J (2024) Diagnostic reference levels in interventional neuroradiology procedures—a systematic review. Neuroradiology 66:2003–2014. 10.1007/s00234-024-03445-5 39243294 10.1007/s00234-024-03445-5 PMC11534899

[40] Valentin J (2000) Avoidance of radiation injuries from medical interventional procedures. Ann ICRP 30:7–67. 10.1016/S0146-6453(01)00004-5 11459599 10.1016/S0146-6453(01)00004-5

[41] Rehani MM, Ciraj-Bjelac O, Vañó E et al (2010) ICRP Publication 117. Radiological protection in fluoroscopically guided procedures performed outside the imaging department. Ann ICRP 40:1–102. 10.1016/j.icrp.2012.03.001. Erratum in: Ann ICRP. 2016;45:351. 10.1177/0146645317705307 10.1016/j.icrp.2012.03.001 22732420

[42] European Commission (2018) European guidelines on diagnostic reference levels for paediatric imaging (Radiation Protection No. 185). Luxembourg: Publications Office of the European Union

[43] European Commission (2018) European guidelines on diagnostic reference levels for paediatric imaging (Radiation Protection No. 199). Luxembourg: Publications Office of the European Union

[44] McParland BJ (1998) A study of patient radiation doses in interventional radiological procedures. Br J Radiol 71:175–185. 10.1259/bjr.71.842.9579182 9579182 10.1259/bjr.71.842.9579182

[45] Brambilla M, Marano G, Dominietto M, Cotroneo AR, Carriero A (2004) Patient radiation doses and references levels in interventional radiology. Radiol Med 107:408–418 15103292

[46] Vano E, Järvinen H, Kosunen A et al (2008) Patient dose in interventional radiology: a European survey. Radiat Prot Dosim 129:39–45. 10.1093/rpd/ncn024 10.1093/rpd/ncn024 18287189

[47] Sandborg M, Rossitti S, Pettersson H (2010) Local skin and eye lens equivalent doses in interventional neuroradiology. Eur Radiol 20:725–733. 10.1007/s00330-009-1598-9 19727739 10.1007/s00330-009-1598-9

[48] Kien N, Rehel JL, Etard C, Aubert B (2011) Dose patient en neuroradiologie interventionnelle: bilan d’une enquête multicentrique [Patient dose during interventional neuroradiology procedures: results from a multi-center study]. J Radiol 92:1101–1112. 10.1016/j.jradio.2011.08.005 22153042 10.1016/j.jradio.2011.08.005

[49] Sailer AM, Paulis L, Vergoossen L et al (2017) Real-time patient and staff radiation dose monitoring in IR practice. Cardiovasc Interv Radiol 40:421–429. 10.1007/s00270-016-1526-8 10.1007/s00270-016-1526-8 PMC5288431 27942927

[50] Pace E, Cortis K, Debono J, Grech M, Caruana CJ (2020) Establishing local and national diagnostic and interventional cardiology and radiology reference levels in a small European state: the case of Malta. Radiat Prot Dosim 191:261–271. 10.1093/rpd/ncaa152 10.1093/rpd/ncaa152 33094323

[51] Vano E, Fernandez JM, Sanchez RM et al (2013) Patient radiation dose management in the follow-up of potential skin injuries in neuroradiology. AJNR Am J Neuroradiol 34:277–282. 10.3174/ajnr.A3211 22859286 10.3174/ajnr.A3211 PMC7965086

[52] Borota L, Jangland L, Åslund PE et al (2017) Spot fluoroscopy: a novel innovative approach to reduce radiation dose in neurointerventional procedures. Acta Radiol 58:600–608. 10.1177/0284185116658682 27522095 10.1177/0284185116658682 PMC5347367

