# Supplementary material for: Diagnostic reference levels in interventional neuroradiology: a scoping review
Source: Eur Radiol. 2026 Mar 25;36(8):6599–611. doi: 10.1007/s00330-026-12472-0 (PMC13342147; doi:10.1007/s00330-026-12472-0)
Supplement: Supplementary file 1 — ELECTRONIC SUPPLEMENTARY MATERIAL [file 330_2026_12472_MOESM1_ESM.pdf]

Diagnostic Reference Levels (DRLs) in Interventional Neuroradiology (INR): A Scoping Review

ELECTRONIC SUPPLEMENTARY MATERIAL

Supplementary material table 1: Bolean combinations used for literature search

**INR:**
("coiling" OR "embolisation" OR "embolization" OR "endovascular treatment" OR "endovascular therapy" OR AIS OR stroke OR "cerebral ischaemia" OR “cerebral ischemia” "cerebral infarction" OR “aneurysm” OR “AVM” OR “AVF” OR "arteriovenous malformation" OR “thrombectomy” OR “embolectomy” OR “revascularization” OR “stentriever” OR “treatment” OR “mechanical” OR “aspiration” OR “DSA” OR “angiography” OR “angiogram” OR “arteriography” OR “INR” OR “neuro intervention” OR "endovascular neurology" OR "endovascular neurosurgery" OR "interventional radiology")
**DRL:**
(DAP OR "dose area product" OR "fluoroscopy time" OR "cumulative air Kerma" OR "diagnostic reference level" OR “DRL” OR "dose reference level" OR "reference levels" OR “RL”)
**Optimisation:**
("image receptor" OR "last image hold" OR "pulsed fluoroscopy" OR "spectral filtration" OR collimation OR "personnel training" OR “noise” OR “optimization” OR “optimisation” OR "image quality" OR "patient radiation dose" OR "radiographic image enhancement" OR "digital radiography" OR "optimal exposure")

Supplementary material table 2: Eligibility criteria

Inclusion Criteria
Studies on Interventional Neuroradiological procedures
Studies on optimization of Interventional Neuroradiological procedures
Studies providing information on DRLs and their establishment in Interventional Neuroradiological procedures
Both prospective and retrospective studies
Age of participants > 18 years
Including phantom studies
Including animal studies
Studies available in the English Language
No date restriction
**Exclusion Criteria**
Age of participants < 18 years
Studies not available in the English Language
